# Pathological Signal‐Responsive Nanoplatforms for Sepsis: Integrating Biomarker Sensing With Spatiotemporal Drug Delivery and Immunomodulation

**DOI:** 10.1002/advs.77718

**Published:** 2026-09-16

**Authors:** Yukun Liu, Kang Wang, Kwet Kyawl Yan, Zhikai Xu, Xuan Zhao, Xiangjun Bai, Zhanfei Li, Yuchang Wang

**Affiliations:** ^1^ Department of Plastic and Aesthetic Surgery Tongji Hospital Tongji Medical College Huazhong University of Science and Technology Wuhan China; ^2^ The Second Clinical College Tongji Medical College Huazhong University of Science and Technology Wuhan China; ^3^ Division of Trauma Surgery Emergency Surgery & Surgical Critical Tongji Hospital Tongji Medical College Huazhong University of Science and Technology Wuhan China; ^4^ Trauma Center Tongji Hospital Tongji Medical College Huazhong University of Science and Technology Wuhan China; ^5^ Department of Emergency and Critical Care Medicine Tongji Hospital Tongji Medical College Huazhong University of Science and Technology Wuhan China; ^6^ Sino‐German Research Institute of Disaster Medicine Wuhan China

**Keywords:** drug delivery, precision medicine in sepsis, sepsis, stimulus‐responsive drug delivery, stimulus‐responsive nanoparticle

## Abstract

Sepsis remains a life‐threatening syndrome characterized by dynamic immune dysregulation and multi‐organ failure. Stimulus‐responsive nanoplatforms, which function as “biomarker‐triggered” therapeutic systems, have emerged as a frontier in precision sepsis management. These platforms are engineered to autonomously sense pathological hallmarks within the sepsis microenvironment—such as fluctuations in pH, reactive oxygen species (ROS), and specific enzymatic over‐expression—thereby enabling precise, spatiotemporal drug release. Unlike conventional static therapies, these “smart” nanoplatforms modulate therapeutic intervention in real‐time based on disease progression. This review comprehensively evaluates the integration of sensing and therapy using nanoplatforms loaded with antibiotics, antimicrobial peptides, anti‐inflammatory agents, and gene‐regulatory molecules. We highlight how these platforms optimize therapeutic windows by enhancing local drug bioavailability while minimizing systemic off‐target toxicity. Furthermore, we discuss the current hurdles in clinical translation, including patient‐specific biomarker variability and the complexity of scalable fabrication. Finally, we propose future directions, emphasizing the synergy of real‐time biosensing, artificial intelligence (AI), and multimodal “theranostic” platforms to achieve stage‐specific, personalized interventions. These advancements hold the potential to redefine sepsis management by shifting from “one‐size‐fits‐all” treatments to biomarker‐driven precision medicine.

## Graphical abstract

Biomarker‐triggered nanoplatforms transform sepsis therapy by coupling pathological sensing with precision drug release. This review reveals how microenvironment‐responsive nanomedicines enable stage‐specific intervention, improve therapeutic efficacy, and minimize systemic toxicity, while highlighting emerging opportunities for AI‐assisted and multimodal theranostic strategies.

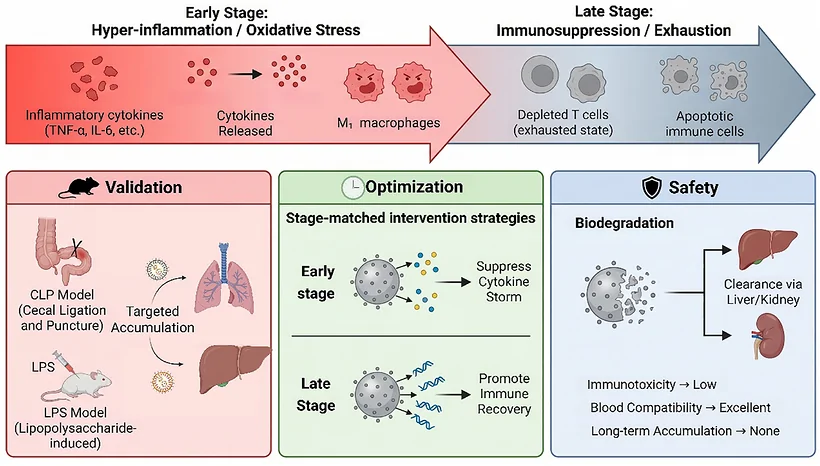

## 1 Introduction

Sepsis is a life‐threatening systemic syndrome triggered by infection and fundamentally characterized by a profound dysregulation of host immune responses. It remains one of the leading causes of mortality and long‐term functional impairment in intensive care units worldwide, accounting for approximately 20 million deaths annually and contributing to 1 in 5 deaths globally [1, 2]. The global incidence of sepsis is estimated at 49 million cases per year (Sepsis‐3 definition), with hospital mortality rates ranging from 25% to 50% depending on disease severity (sepsis vs. septic shock) [3]. Furthermore, approximately 30–50% of sepsis survivors experience long‐term functional impairment, including cognitive deficits, neuromuscular dysfunction, and reduced quality of life — a constellation of symptoms increasingly recognized as post‐sepsis syndrome [4]. Contrary to the traditional view that sepsis represents a unidirectional process of uncontrolled inflammation, accumulating evidence indicates that sepsis is a highly dynamic and spatiotemporally heterogeneous immunopathological disorder rather than a simple hyperinflammatory state [5, 6]. During disease progression, patients often experience overlapping or sequential phases of cytokine storm, immune suppression, and multiple organ dysfunction. These intertwined and continuously evolving pathological processes substantially complicate therapeutic decision‐making and contribute to the marked heterogeneity of clinical outcomes in sepsis [7]. From an immunopathological perspective, the early phase of sepsis is typically dominated by an exaggerated pro‐inflammatory response, characterized by the excessive release of cytokines and chemokines. This inflammatory amplification disrupts endothelial barrier integrity, impairs microcirculatory perfusion, and promotes widespread tissue injury [8, 9]. In contrast, a considerable proportion of patients rapidly transition into a state of immune paralysis at later stages, marked by functional exhaustion of immune cells, impaired antigen presentation, and the development of acquired immunosuppression. This immunosuppressive phenotype markedly increases susceptibility to secondary infections and is closely associated with adverse prognosis and mortality [10, 11]. Collectively, these observations underscore that sepsis is essentially a disease of failed immune homeostasis. Accordingly, effective therapeutic strategies should extend beyond pathogen eradication or indiscriminate inflammation suppression, and instead aim to restore a balanced and adaptive immune response.

Despite this evolving understanding, current clinical management of sepsis still relies predominantly on broad‐spectrum antibiotics combined with supportive care for organ dysfunction [12, 13]. Although antimicrobial therapy is indispensable for controlling the primary infection, its therapeutic targets are largely confined to pathogens and do not directly address the underlying immune dysregulation that drives disease progression [14]. Meanwhile, numerous anti‐inflammatory interventions targeting individual cytokines or signaling pathways have failed to demonstrate consistent clinical benefit in large‐scale trials, with some studies even reporting an exacerbation of immunosuppression [15, 16]. A fundamental reason for these disappointing outcomes lies in the inability of conventional pharmacological strategies to accommodate the pronounced temporal dynamics and spatial heterogeneity of sepsis. As a result, therapeutic agents are often unable to act at the appropriate site, time window, or intensity required for effective immune modulation [17].

Against this backdrop, nanomedicine has emerged as a promising technological avenue for sepsis therapy [18, 19]. Nanocarrier‐based drug delivery systems can enhance the in vivo stability of therapeutic agents, prolong systemic circulation, and, to some extent, promote their accumulation at inflamed or infected sites, thereby reducing off‐target toxicity and improving therapeutic efficacy [20, 21]. In recent years, a variety of nano‐formulated antibiotics, anti‐inflammatory agents, and immunomodulatory molecules have shown encouraging results in preclinical sepsis models [19, 20, 22]. Nevertheless, it is important to recognize that most existing nanoplatforms still operate in a predominantly “passive delivery” mode, in which drug release profiles are predefined and largely insensitive to the rapidly changing pathological microenvironment of sepsis [23, 24]. This intrinsic mismatch between static system design and dynamic disease progression has significantly constrained the translational potential of nanomedicine in sepsis. To address these limitations, stimulus‐responsive nanoplatforms have attracted increasing attention [25]. These systems are designed to sense sepsis‐associated pathological cues—such as elevated oxidative stress, pH fluctuations, aberrant enzymatic activity, or pathogen‐derived molecular signals—and to trigger drug release or functional transformation in response to these stimuli [19, 26]. Compared with conventional delivery strategies, stimulus‐responsive nanoplatforms offer a higher degree of spatiotemporal precision and introduce the possibility of dynamically modulating host immune responses [27]. In this context, therapeutic intervention can evolve from a unidirectional approach toward a state‐aware, self‐adaptive regulatory strategy [28].

While nanotechnology‐driven interventions for sepsis have gained substantial traction, delineating the boundaries of this review relative to existing literature is essential. Prior milestone publications have undoubtedly laid a foundational framework. Specifically, Luo et al.^27^provided a macro‐overview of homeostatic reconstruction but relied heavily on static chemistry. Wang et al. [25] conceptualized the hyperinflammation‐to‐immunosuppression transition, yet their discussion remained a brief immunological prospection lacking chemical engineering formulations or in vivo clearance evaluations under multiple organ dysfunction syndrome (MODS). Furthermore, general stimulus‐responsive reviews typically focus on localized inflammation [29] rather than the systemic, hyper‐dynamic cascades unique to sepsis, while contemporary overviews integrating artificial intelligence (AI) or CRISPR [30] tend to enumerate technologies in a “collage‐style” rather than offering a mechanistic critique of self‐adaptive execution.

To transcend these incremental summaries, this review establishes a distinct paradigm. We explicitly bridge the gap between materials engineering and host immunometabolic reprogramming, evaluating how multi‐stimuli responsive nanoplatforms function as autonomous, state‐aware regulators. Rather than replicating case‐by‐case single successes, we systematically contrast host‐derived pathological cues with infection‐specific exogenous stimuli to construct a comprehensive logic‐gate evaluation matrix (summarized in Table 1). Crucially, we present a forward‐looking critique on integrating real‐time in vivo biosensing with closed‐loop AI logical feedback arrays for endotype‐matched precision interventions—a translational roadmap missing in prior literature. This granular analysis provides a definitive reference to accelerate the clinical translation of smart nanomedicines from benchmarking laboratories to the intensive care unit.

**TABLE 1:** Molecular design principles of stimulus‐responsive nanoplatforms based on sepsis‐associated pathological signals.

Sepsis‐associated factor	Specific functional groups / smart linkages	Molecular triggering mechanism	Representative platforms	References
**Mild acidity** (pH 6.5–6.8)	Hydrazone, acetal, and ketal bonds Imidazole‐containing frameworks (e.g., ZIF‐90) Poly(2‐(diisopropylamino)ethyl methacrylate) (PDPA)	Hydronium ion‐catalyzed hydrolysis of acid‐labile bonds Protonation‐induced framework dissociation or structural collapse Charge reversal‐mediated hydrophobic‐to‐hydrophilic phase transition	β‐CD‐PDPA/DSPE‐PEG micelles MI@ZIF‐90 nanoplatforms pH‐responsive GLUT1 inhibitor delivery systems	[68, 69, 70]
**Elevated ROS** (H_2_O_2_, ·OH)	Thioketal (TK) linkers Thioether groups Boronic acid esters Selenium‐containing bonds	ROS‐triggered oxidative cleavage of thioketal or boronate structures Oxidation‐induced transformation of hydrophobic sulfide groups into hydrophilic sulfoxide/sulfone structures Oxidative stress‐mediated carrier disassembly and drug release	Atv/PTP‐TCeria nanoparticles with mPEG‐TK‐PLGA coating DMSNM@C‐178/PAA platform	[70, 79]
**Aberrant enzymes** (MMPs, lipases, hyaluronidase)	Enzyme‐cleavable peptide sequences (e.g., PLGLAG) Ester bonds and phospholipid‐based matrices Ascorbyl stearate (AS)‐derived functional motifs	Sequence‐specific proteolytic cleavage mediated by MMPs Enzyme‐catalyzed degradation of lipid matrices Competitive substrate interaction with bacterial‐associated enzymes	VCM‐AS‐SLNs lipid nanoparticles targeting bacterial lipase and hyaluronidase activity	[86]
**Microbial stimuli** (LPS, cf‐mtDNA)	Electrically neutral phosphocholine ligands Cationic recognition domains Recombinant deoxyribonuclease I (DNase I)	Charge rearrangement and dipole‐mediated recognition of lipid A in LPS Enzymatic hydrolysis of cell‐free mitochondrial DNA Pathogen‐associated molecular pattern recognition and neutralization	PCAPAN‐Fe magnetic nanoadsorbents for LPS removal DNase I/HSA hybrid protein nanomotors	[92, 93]
**Exogenous physical stimuli** (Ultrasound)	Phase‐change contrast agents (e.g., perfluoropentane, PFP) Cavitation‐sensitive lipid bilayers	Ultrasound‐induced cavitation effects Mechanical disruption of lipid bilayers causing transient pore formation and controlled payload release	LIPs‐S@TAK/PFP serine‐modified liposomes FOT/Fe_3_O_4_@Lipo‐ICG liposomes	[98, 99]

Accordingly, stimulus‐responsive nanoplatforms are increasingly regarded as a key enabling technology for stage‐specific and personalized sepsis therapy [29]. By integrating pathological signal recognition, controlled drug delivery, and immune modulation within a single nanosystem, these platforms hold the potential to suppress excessive inflammation while avoiding overt immunosuppression, thereby facilitating the restoration of immune homeostasis [30]. In this review, we critically summarize recent advances in stimulus‐responsive nanoplatforms for sepsis therapy, with a particular focus on their design principles, stimulus‐triggering mechanisms, and immunomodulatory functions, aiming to provide a conceptual framework for future basic research and clinical translation.

## 2 Dynamic Pathological Microenvironment of Sepsis: Biological Basis for Stimulus‐Responsive Design

**FIGURE 1:**
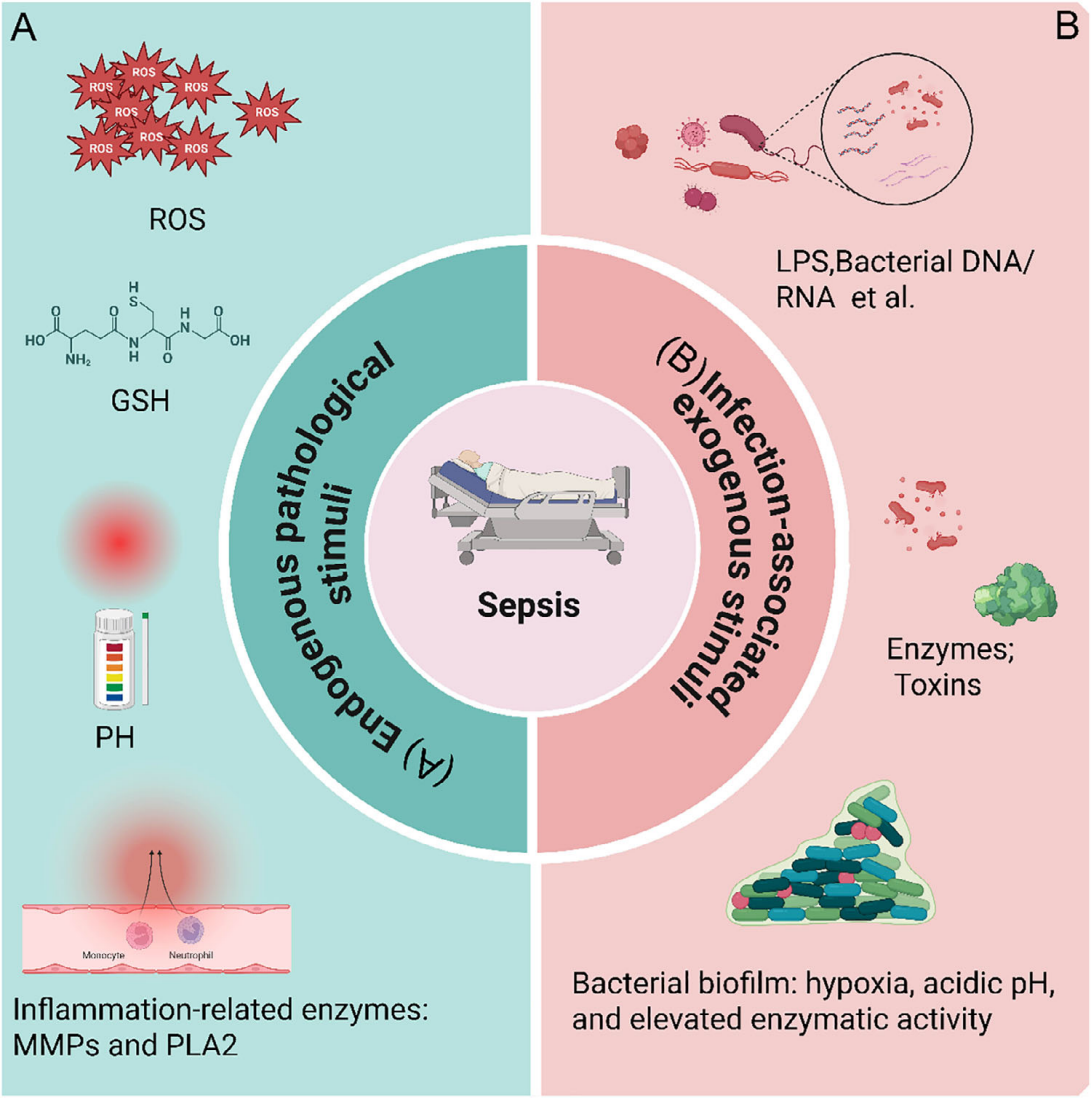
Endogenous pathological and infection‐associated exogenous stimuli in sepsis. Sepsis features a highly dynamic microenvironment shaped by endogenous pathological stimuli and infection‐associated exogenous cues. (A) Endogenous stimuli include excessive reactive oxygen species (ROS), glutathione (GSH) depletion, pH dysregulation, and elevated inflammation‐related enzymes such as matrix metalloproteinases (MMPs) and phospholipase A2 (PLA2). (B) Exogenous stimuli arise from invading pathogens and their products, including lipopolysaccharide (LPS), bacterial DNA/RNA, secreted enzymes, and toxins. Within bacterial biofilms, hypoxia, acidic pH, and enhanced enzymatic activity further aggravate local inflammation and immune dysfunction. Together, these stimuli define the sepsis microenvironment and represent key targets for precision nanotherapeutic intervention. (Created with BioRender.com).

Stimulus‐responsive nanomedicine relies on the existence of sharp physiological contrasts between healthy tissues and diseased sites. In the context of sepsis, this contrast is exceptionally pronounced, manifested as a “storm” of oxidative stress, pH fluctuations, and enzymatic dysregulation. These pathological hallmarks serve as reliable endogenous and exogenous “triggers” for the selective activation of smart nanocarriers. To provide a rational basis for such responsive designs, this section systematically analyzes the septic microenvironment across three dimensions: host‐derived pathological stimuli, infection‐specific cues, and the temporal evolution of these signals throughout the disease course (Figure 1).

### 2.1 Sepsis‐Associated Pathological Microenvironmental Stimuli

During sepsis, infection‐induced immune activation and profound metabolic dysregulation jointly give rise to a highly aberrant endogenous pathological microenvironment, providing multidimensional and readily perceivable biological cues for the design of stimulus‐responsive nanoplatforms. Among these features, oxidative stress represents one of the most prominent hallmarks of sepsis [31, 32]. Excessive activation of neutrophils, macrophages, and endothelial cells leads to sustained overproduction of reactive oxygen and nitrogen species (ROS/RNS), which accumulate at sites of infection and organ injury. Physicochemically, while extracellular hydrogen peroxide (H_2_O_2_) concentrations are tightly maintained within a low nanomolar threshold (<100 nM) in healthy tissues, a localized respiratory burst during acute sepsis causes these values to surge abruptly into a micromolar window of 10–100 µM at primary infection foci [33]. This pathological elevation in oxidative stress not only drives lipid peroxidation, protein oxidation, and DNA damage, thereby exacerbating tissue dysfunction, but also establishes distinct spatial gradients, rendering it a highly representative endogenous stimulus for responsive nanomedicine [34, 35].

Concurrently, microcirculatory dysfunction and alterations in cellular metabolic programs result in local tissue ischemia and hypoxia, which promote enhanced glycolysis and lactate accumulation. Consequently, infected and injured microenvironments exhibit a pronounced decline in extracellular pH, dropping from the homeostatic level of 7.4 to a localized acidic range of 6.5–6.8 [36, 37]. This decreased pH not only further modulates immune cell function and drug activity, but also provides a rational basis for the selective activation of pH‐sensitive nanostructures [38]. In addition to oxidative and acidic alterations, sepsis is accompanied by widespread disruption of intracellular and extracellular redox homeostasis. The synthesis, consumption, and spatial distribution of reducing molecules, particularly glutathione (GSH), are markedly altered. Intracellular GSH, which typically ranges from 2 to 10 mM under standard physiology, undergoes severe consumption and dynamic fluctuations across different organs and immune cell subsets due to over‐activated scavenging pathways [39]. This imbalance in reductive capacity creates favorable conditions for nanoplatforms incorporating reducible chemical linkages to achieve cell‐ or lesion‐specific payload release. Moreover, a variety of inflammation‐associated enzymes are aberrantly activated during sepsis, including matrix metalloproteinases (MMPs), phospholipase A2 (PLA2), and multiple proteases. Specifically, transcriptional and post‐translational upregulation drives a 5–10‐fold increase in matrix metalloproteinase‐2/9 (MMP‐2/9) activity above basal levels, accompanied by robust degranulation of recruited neutrophils, which elevates local neutrophil elastase (NE) concentrations beyond a critical pathological threshold of 1 µg mL^−^^1^ [40, 41, 42, 43]. Collectively, these endogenous stimuli rarely exist in isolation; instead, they coexist and interact within the septic microenvironment, forming a highly dynamic and spatially heterogeneous stimulus network. This complex landscape provides a robust biological foundation for stimulus‐responsive nanoplatforms capable of precise sensing and selective activation (Figure 1A).

### 2.2 Infection‐Associated Stimuli in Sepsis

Beyond host‐derived pathological alterations, infection itself introduces a distinct class of exogenous stimuli that serve as highly specific triggers within the septic microenvironment. Among these, pathogen‐associated molecular patterns (PAMPs) are the most representative infection‐related signals, including lipopolysaccharide (LPS) from Gram‐negative bacteria, lipoteichoic acid (LTA) from Gram‐positive bacteria, and β‐glucans from fungal cell walls [44]. These molecules directly engage pattern recognition receptors, such as Toll‐like receptors, thereby driving innate immune activation and inflammatory cascades. Their presence and abundance can, to some extent, reflect pathogen burden and infection activity in vivo [45]. Stimulus‐responsive strategies based on PAMP recognition enable nanoplatforms to acquire the capability of “responding upon pathogen detection,” thereby achieving enhanced therapeutic selectivity at infection‐associated sites. In addition to structural components, metabolically active pathogens secrete a wide array of enzymes and toxins that participate in tissue destruction, immune evasion, and inflammatory dissemination. These secreted virulence factors are typically enriched at infection foci and dynamically fluctuate with microbial activity, providing a critical exogenous basis for the development of infection‐activity–dependent responsive systems [46, 47]. Furthermore, in certain sepsis and secondary infection scenarios, pathogens can form biofilms, within which a complex microenvironment characterized by hypoxia, mild acidity, elevated enzymatic activity, and metabolic heterogeneity is established [48]. Biofilm formation not only markedly enhances bacterial tolerance to antibiotics and host immune defenses, but also offers a clinically relevant context for the design of nanoplatforms capable of synergistically responding to multiple stimuli [49, 50]. Taken together, these infection‐derived exogenous signals intertwine with host endogenous pathological cues to shape the distinctive septic infection microenvironment, conferring unique advantages to stimulus‐responsive nanoplatforms for infection‐specific recognition and precision therapy (Figure 1B).

### 2.3 Association Between Stimulus Signals and Sepsis Stages

It is important to emphasize that neither endogenous nor exogenous stimulus signals in sepsis remain constant throughout the disease course. Instead, they exhibit pronounced stage‐dependent dynamics in response to infection progression and evolving host immune status [51]. In the early phase of sepsis, infection‐triggered hyperactivation of innate immunity frequently culminates in a cytokine storm, characterized by abrupt surges in pro‐inflammatory cytokines and chemokines, accompanied by intense oxidative stress mediated by neutrophils and macrophages [52]. During this stage, ROS/RNS levels rise dramatically and synergize with cytokine cascades to amplify endothelial injury, increase vascular permeability, and precipitate organ dysfunction, rendering oxidative stress and inflammation‐associated cues the dominant pathological signals [52, 53].

As sepsis advances, a subset of patients gradually transitions into a phase dominated by immunosuppression. This stage is characterized by metabolic reprogramming defects, sustained redox imbalance, and impairment of adaptive immune responses, most notably T‐cell exhaustion and diminished antigen responsiveness [7, 54]. Under such conditions, interventions solely targeting inflammatory signals often fail to improve outcomes and may even exacerbate immune dysfunction [7]. These stage‐dependent shifts underscore the highly dynamic and spatiotemporally heterogeneous nature of stimulus signals within the septic microenvironment, with different cues predominating at different time points. In clinical reality, sepsis rarely presents as a strictly chronological transition from hyperinflammation to immunosuppression; instead, these phenotypes often overlap dynamically or coexist within individual patients [55]. To guide the deployment of stage‐matched nanomedicines, clinical staging must rely on measurable, quantifiable biomarker panels rather than rigid timelines. Hyperinflammatory endotypes are typically characterized by high circulating levels of C‐reactive protein (CRP), procalcitonin (PCT), interleukin‐6 (IL‐6), and tumor necrosis factor‐alpha (TNF‐α) [55]. Conversely, the transition to, or coexistence of, an immunosuppressive state can be monitored through the downregulation of human leukocyte antigen‐DR (HLA‐DR) expression on monocytes (<30\%), increased percentages of regulatory T cells (Tregs) or myeloid‐derived suppressor cells (MDSCs), and elevated concentrations of soluble programmed death‐ligand 1 (sPD‐L1) [56]. Utilizing these quantifiable immunoassay panels and flow cytometry profiles allows clinicians to stratify patient endotypes in real‐time, providing explicit criteria for smart nanoplatforms to adaptively execute anti‐inflammatory or immunostimulatory protocols [57].

To balance these temporal and spatial fluctuations in biomarker concentrations during platform development, nanomedicines must transition from rigid, single‐threshold triggers to adaptive architectures. Materials engineers can balance these fluctuations through three primary strategies: (1) implementing boolean logic‐gate networks (e.g., dual pH/ROS “AND” gates) to prevent off‐target systemic leakage; (2) performing chemical threshold tuning by modulating linkage density or hydrophobic/hydrophilic ratios to calibrate activation sensitivities to specific disease phases; and (3) designing cascaded core‐shell structures that enable sequential outer‐to‐inner disassembly synchronizing with the shifting immunopathological landscape. Integrating these responsive chemistries allows nanoplatforms to function as state‐aware, self‐adaptive interventions.

Stimulus‐responsive nanodelivery systems can generally be categorized according to the origin of the triggering signals into endogenous stimulus–driven systems (pathological microenvironment–responsive) and exogenous stimulus–activated systems (externally controlled by physical signals). Importantly, both endogenous pathological alterations and exogenous infection‐associated signals converge to create the aberrant microenvironment that stimulus‐responsive nanoplatforms are designed to detect and exploit. This intrinsic complexity fundamentally explains why sepsis treatment cannot achieve durable efficacy through single‐target or static therapeutic strategies, while simultaneously providing a strong theoretical rationale for stimulus‐responsive nanoplatforms capable of sensing pathological states and adjusting therapeutic behavior accordingly [58]. By integrating multiple stimulus signals into identifiable response triggers, stimulus‐responsive nanosystems hold promise for implementing disease stage–matched, self‐adaptive interventions. Such approaches may suppress excessive inflammation while avoiding further impairment of immune function, ultimately facilitating the restoration of immune homeostasis.

## 3 Design Principles and Response Mechanisms of Stimulus‐Responsive Nanoplatforms

**FIGURE 2:**
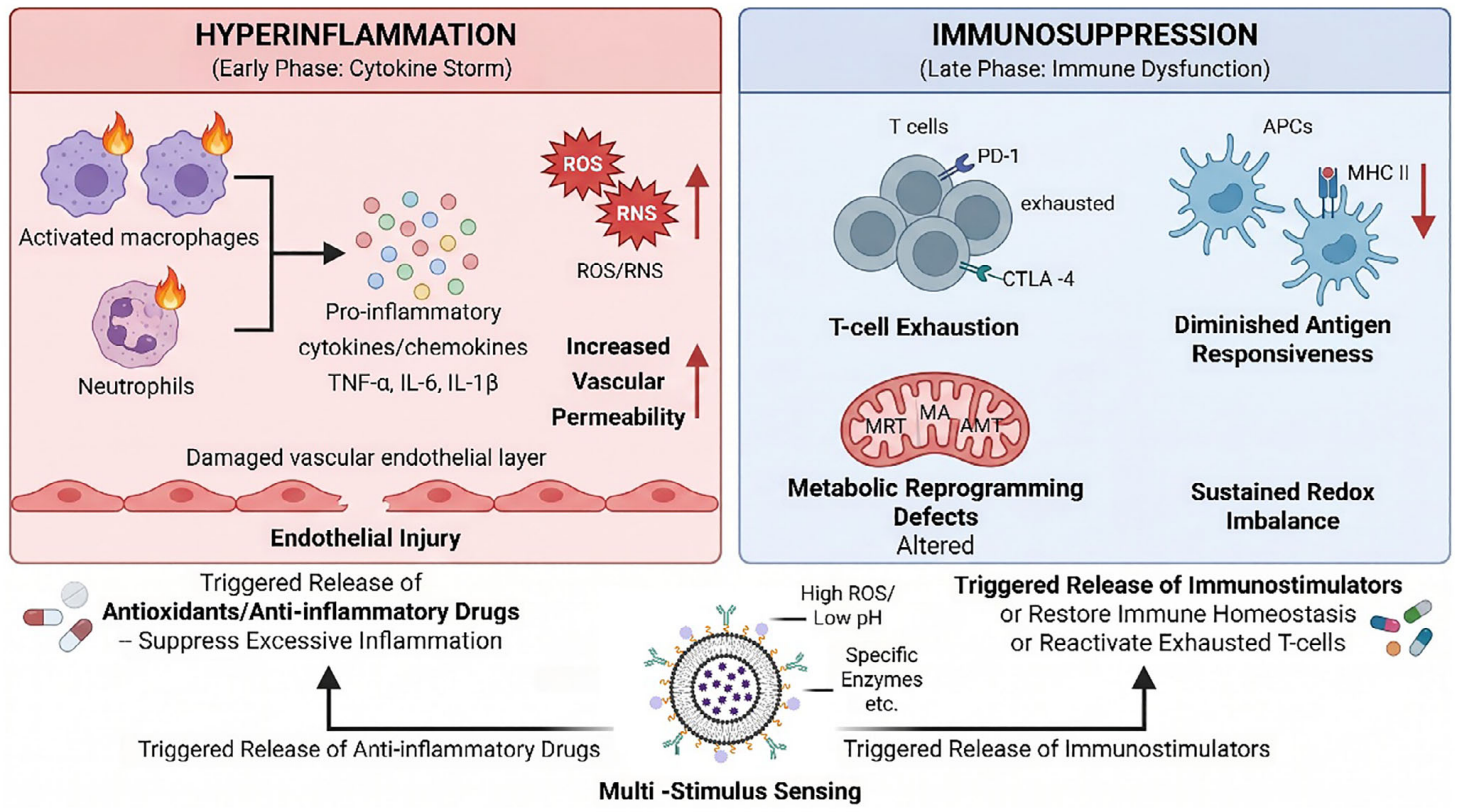
Orchestrating stage‐specific sepsis therapy via multi‐stimulus responsive nanomedicine. Sepsis progression is characterized by a biphasic immune response. (Left) During the early hyperinflammation phase (cytokine storm), activated macrophages and neutrophils release excessive pro‐inflammatory cytokines and ROS/RNS, leading to vascular endothelial injury. (Right) The late immunosuppression phase is marked by T‐cell exhaustion (upregulation of PD‐1/CTLA‐4), diminished antigen responsiveness in APCs, and metabolic reprogramming defects. (Bottom) A multi‐stimulus sensing nanoplatform is designed to synchronize drug release with these pathophysiological stages: triggering the release of antioxidants and anti‐inflammatory drugs to suppress early‐phase inflammation, while delivering immunostimulators to reactivate exhausted immune cells and restore homeostasis during the late phase.(Created with BioRender.com).

Stimulus‐responsive nanodelivery systems can generally be categorized according to the origin of the triggering signals into endogenous stimulus–driven systems (pathological microenvironment–responsive) and exogenous stimulus–activated systems (externally controlled by physical signals). Endogenous‐responsive nanoplatforms rely on intrinsic features of the infectious or inflammatory microenvironment to achieve selective drug release without the need for external intervention, making them particularly suitable for systemic infectious diseases such as sepsis. In contrast, exogenous‐responsive systems employ external triggers—including light, magnetic fields, or ultrasound—to enable highly controllable drug delivery; however, their application in systemic sepsis remains limited by challenges related to spatial accessibility, tissue penetration, and clinical operability (Figure 2). A primary challenge in engineering these nanoplatforms is balancing circulatory stability with pathological sensitivity. To prevent premature drug leakage under standard physiology (pH 7.4), carriers require stable structures, often achieved via dense crosslinking or hydrophobic cores. However, over‐stabilization can blunt release kinetics at the target site. To overcome this stability‐sensitivity trade‐off, recent designs utilize cooperative, multi‐valent, or non‐linear linkages. These chemical switches remain dormant during normal systemic circulation but trigger an abrupt, “all‐or‐nothing” disassembly once microenvironmental parameters cross critical pathological thresholds. Calibrating this sharp kinetic transition is essential to maximize localized efficacy while eliminating off‐target systemic toxicity.

### 3.1 Endogenous Stimuli

**FIGURE 3:**
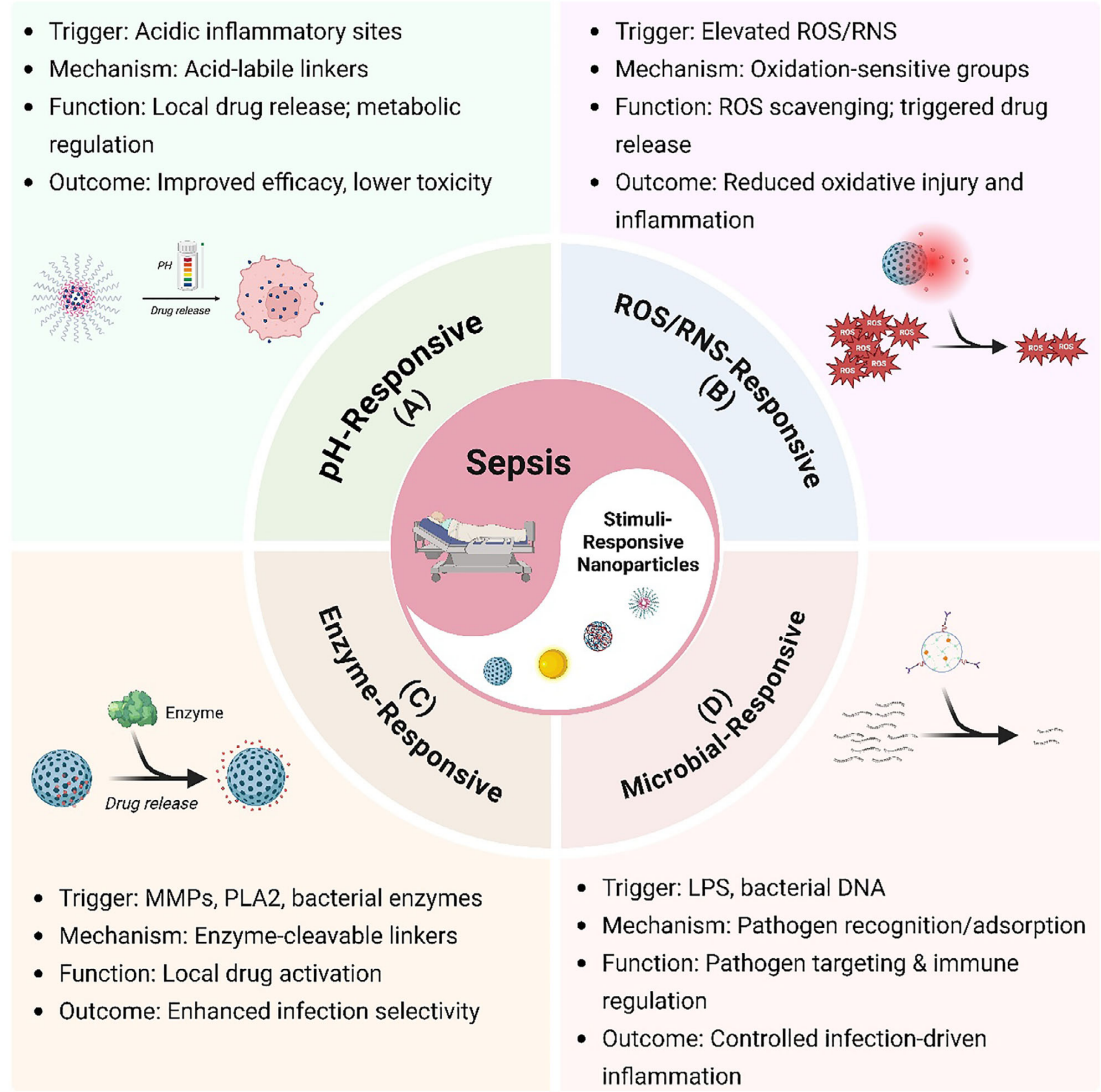
Stimuli‐responsive nanoparticle strategies for precision therapy in sepsis. Representative stimuli‐responsive nanoparticle systems designed to sense and respond to the sepsis microenvironment are illustrated. (A) pH‐responsive nanoparticles enable localized drug release at acidic inflammatory sites via acid‐labile linkers. (B) ROS/RNS‐responsive nanoparticles employ oxidation‐sensitive moieties to scavenge reactive species and trigger drug release. (C) Enzyme‐responsive nanoparticles are activated by inflammation‐ or pathogen‐associated enzymes, including MMPs, PLA2, and bacterial enzymes, allowing site‐specific drug activation. (D) Microbial‐responsive nanoparticles recognize pathogen‐associated molecular patterns, such as LPS and bacterial DNA, to achieve targeted pathogen interaction and immune modulation. Collectively, these platforms enable spatiotemporally controlled drug delivery and improved therapeutic precision in sepsis. (Created with BioRender.com).

Endogenous stimuli arise from the host's distinct pathophysiological shifts during sepsis, including localized acidification, oxidative stress, and aberrant enzymatic profiles. By exploiting these intrinsic cues as molecular “switches,” stimulus‐responsive nanoplatforms can execute autonomous, site‐specific drug release. This “on‐demand” activation mechanism bridges the gap between static drug delivery and the dynamic sepsis microenvironment, enhancing therapeutic precision while safeguarding systemic immune integrity. Among these pathological hallmarks, pH fluctuations represent a primary and highly exploitable trigger for localized intervention (Figure 3). To establish chemical specificity toward these distinct cues, nanoplatforms integrate tailored functional groups that undergo predictable chemical transformations [59]. For instance, acid‐labile linkages (e.g., hydrazone, acetal, and ketal bonds) are selectively cleaved via hydronium‐catalyzed hydrolysis in mildly acidic environments (pH 6.5–6.8), while protonatable tertiary amines achieve pH‐responsiveness through charge reversal [60]. Similarly, oxidative stress triggers specific hydrophobic‐to‐hydrophilic transitions or cleavage in boronic acid esters, thioethers, and thioketals upon exposure to micromolar hydrogen peroxide, whereas disulfide and tetrasulfide bonds rely on thiol‐disulfide exchange for reduction‐responsive activation [61]. For enzymatic and microbial targets, specificity is governed by cleavable short peptide sequences (e.g., PLGLAG for MMPs) and pathogen‐targeted affinity ligands like zinc(II)‐dipicolylamine or cationic domains that selectively engage bacterial lipopolysaccharides [62] (Table 1).

#### 3.1.1 PH‐Responsive Nanoplatforms

Among various stimulus‐responsive strategies, pH‐responsive nanoplatforms represent one of the most mature and pathophysiologically relevant systems for sepsis therapy. Their design is fundamentally based on the localized acidic microenvironment commonly observed in infected and injured tissues [63, 64]. During sepsis, heightened bacterial metabolism, enhanced glycolysis in inflammatory cells, and ischemia–hypoxia caused by microcirculatory dysfunction collectively promote lactate accumulation and a decline in local pH. This acidification is spatially confined and markedly distinct from that of healthy tissues, thereby providing a reliable pathological contrast for selective activation [65]. Exploiting this feature, pH‐responsive nanoplatforms incorporate acid‐labile chemical linkages or structural motifs that undergo controlled dissociation or conformational changes under mildly acidic conditions, triggering site‐specific drug release (Figure 3A).

**FIGURE 4:**
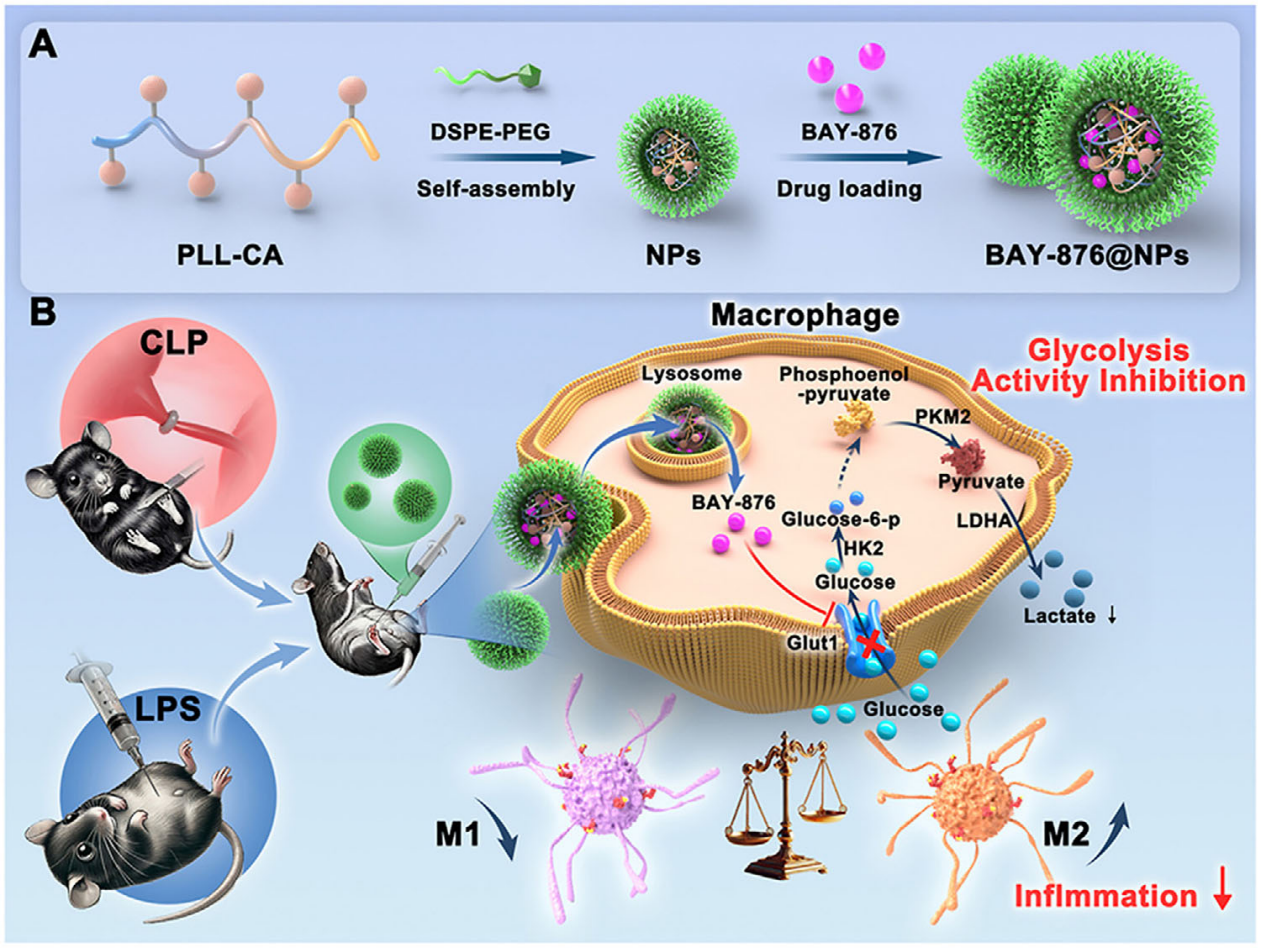
(A) Preparation of BAY‐876@NPs. (B) Schematic illustration of the mechanism by which BAY‐876@NPs inhibit macrophage glycolysis to regulate macrophage polarized and inhibit inflammation to alleviate sepsis. Reproduced with permission [67]. Copyright 2025, Elsevier.

From a materials design perspective, acid‐sensitive linkers such as hydrazone, acetal, and ketal bonds are widely employed to construct pH‐responsive carriers [63]. These linkages remain relatively stable under physiological conditions but readily cleave in acidic environments, enabling selective disassembly or payload release at infection or inflammation sites. By rationally tuning the type, density, and distribution of acid‐sensitive bonds, drug release kinetics and activation thresholds can be finely adjusted to better match the pathological microenvironment [63, 66]. Beyond conventional antimicrobial or anti‐inflammatory delivery, pH‐responsive nanoplatforms have recently been extended to the targeted modulation of immunometabolic pathways. For example, Ni Ding and colleagues developed a pH‐responsive nanoparticle system based on FDA‐approved materials, in which drug release was selectively triggered by the acidic inflammatory microenvironment to precisely modulate macrophage glycolytic metabolism. Under acidic conditions, protonation‐induced disassembly enabled the release of a glucose transporter 1 (GLUT1) inhibitor, thereby suppressing inflammation‐associated glucose uptake and glycolytic flux, reducing lactate accumulation, and reshaping macrophage metabolic phenotypes. This metabolic reprogramming attenuated pro‐inflammatory M1 polarization while promoting an anti‐inflammatory M2 phenotype, ultimately alleviating systemic inflammation, reducing multi‐organ damage, and improving survival in septic animal models. This study highlights that pH‐responsive nanoplatforms can function not only as delivery vehicles but also as microenvironment‐triggered regulators of immune metabolism, offering a novel therapeutic paradigm for sepsis [67] (Figure 4).

Tingting Lin et al. reported a pH‐responsive nanodrug delivery system (MI@ZIF‐90, MI@Zeolitic imidazolate framework‐90) designed for controlled release of metronidazole‐targeting molecules. By encapsulating hydrophilic metronidazole within a hydrophobic ZIF‐90 framework, the system exhibited rapid drug release under acidic conditions while maintaining high stability at physiological pH. Protonation‐driven release facilitated efficient intracellular drug delivery, and the combined antibacterial properties of zinc ions and metronidazole produced synergistic effects, including ROS‐mediated bacterial killing and enhancement of host immune responses. In vitro studies demonstrated that MI@ZIF‐90 effectively eliminated multiple pathogenic bacteria at extremely low concentrations and showed potent inhibitory activity against *Aspergillus fumigatus* and *Candida* albicans [68]. Similarly, Xinyu Wang developed a pH‐responsive nanotherapeutic platform (DMSNM@C‐178/PAA) that integrates ROS scavenging with inhibition of the cGAS–STING pathway for anti‐inflammatory therapy. This nanosystem selectively releases its components within the infectious microenvironment: reduced molybdenum‐based polyoxometalates (Mo‐POMs) efficiently neutralize cytotoxic ROS, while C‐178 selectively suppresses cGAS–STING signaling. Through the combined clearance of excessive ROS and modulation of aberrant innate immune activation, DMSNM@C‐178/PAA effectively attenuated inflammation and prevented organ damage in both in vitro and in vivo sepsis models, representing a promising nanotherapeutic strategy for sepsis intervention [69].

Overall, pH‐responsive nanoplatforms transform localized tissue acidification—a ubiquitous and relatively stable pathological hallmark of sepsis—into a practical trigger for precision drug delivery [70]. In sepsis therapy, such systems can enhance antibiotic release at infection sites, thereby increasing local drug concentrations while reducing systemic exposure and toxicity, which may help suppress pathogen growth and limit the emergence of antimicrobial resistance [68, 71, 72]. For anti‐inflammatory or immunomodulatory agents, pH‐triggered release confines therapeutic activity to regions of active inflammation, minimizing nonspecific suppression of systemic immunity [67, 73]. Nevertheless, the reliance on a single stimulus may limit applicability across complex disease stages, motivating the development of multi‐stimuli–integrated and stage‐adaptive nanoplatforms.

#### 3.1.2 ROS/RNS‐Responsive Nanoplatforms

Oxidative stress is one of the most characteristic endogenous features of sepsis pathology, rendering ROS/RNS‐responsive nanoplatforms particularly attractive for precision therapy [74]. Under infection‐ and inflammation‐driven conditions, neutrophils, macrophages, and endothelial cells continuously generate excessive levels of reactive oxygen and nitrogen species, which peak during the cytokine storm phase and accumulate at sites of infection and organ injury. This pathological oxidative stress not only directly mediates tissue damage but also provides a highly disease‐relevant and readily identifiable trigger for responsive nanosystems. From a materials engineering standpoint, ROS/RNS‐responsive nanoplatforms typically incorporate oxidizable or cleavable functional moieties that enable environmental sensing and structural transformation [75]. Common strategies include the use of thioether groups, selenium‐containing bonds, boronate esters, or polysulfide linkages, which remain stable under physiological conditions but undergo oxidation, cleavage, or conformational changes in ROS/RNS‐enriched environments, thereby triggering carrier disassembly or drug release [75]. Through precise control over the chemical properties and spatial distribution of these responsive motifs, nanoplatforms can achieve enhanced selectivity toward regions of elevated oxidative stress (Figure 3B).

**FIGURE 5:**
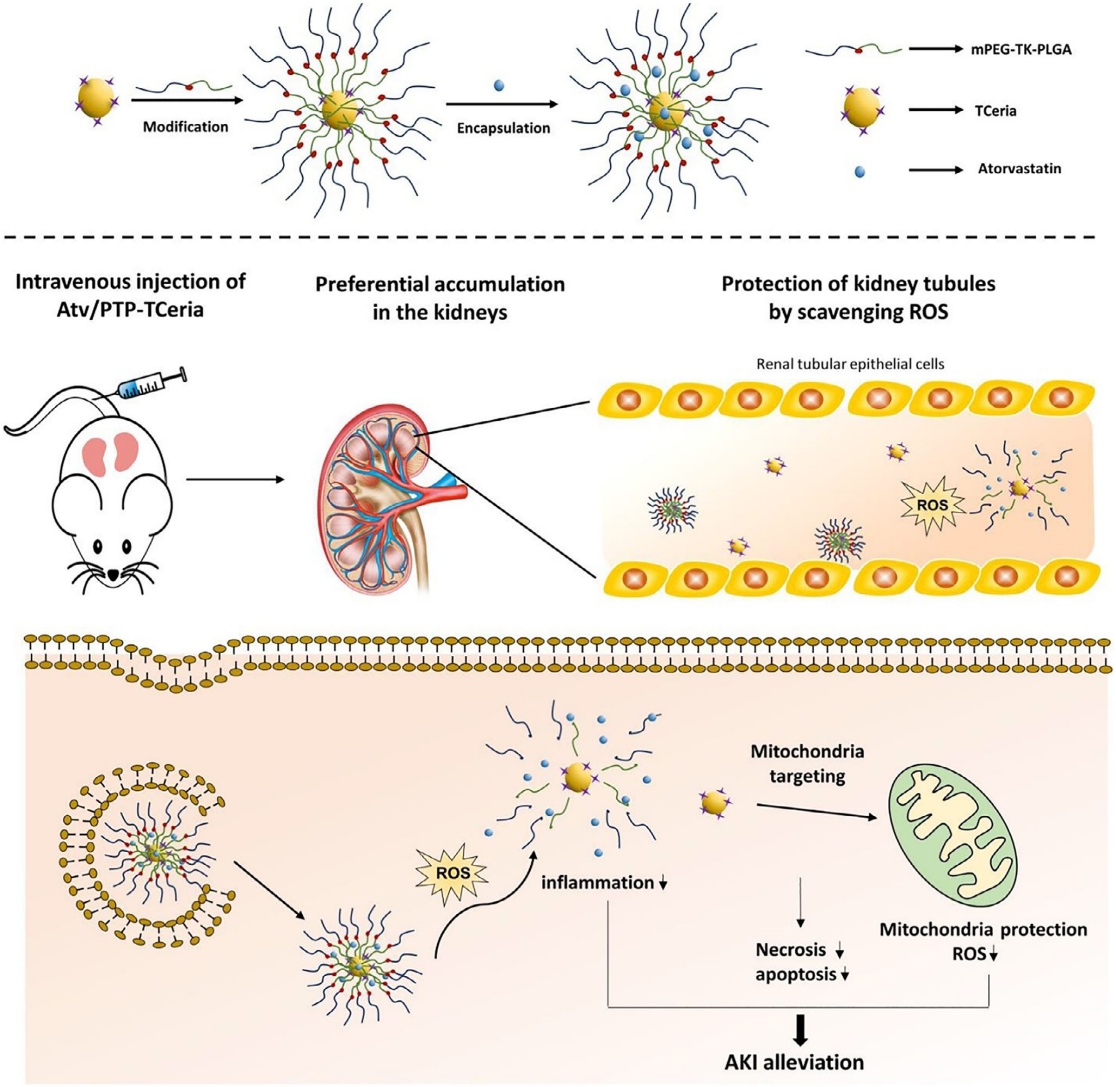
Schematic illustration of Atv/PTP‐TCeria NPs for acute kidney injury. Ceria NPs are modified with triphenylphosphine (TCeria NPs) to be endowed the ability to target mitochondria, then TCeria NPs are coated by mPEG‐TK‐PLGA polymer and load atorvastatin. Atv/PTP‐TCeria NPs could passively target the kidney and release drug responding to high‐level ROS and TCeria NPs would target mitochondria to scavenge excessive ROS for ameliorating AKI. Reproduced with permission [78]. Copyright 2020, Ivyspring International Publisher.

Distinct from passive delivery systems, ROS/RNS‐responsive nanoplatforms in sepsis often exhibit dual “responsive–therapeutic” functionality [76]. On one hand, the carrier matrix itself can consume or scavenge excessive ROS/RNS, alleviating oxidative stress and limiting lipid peroxidation–induced cellular injury. On the other hand, oxidation‐triggered release of anti‐inflammatory, antioxidant, or immunomodulatory agents further suppresses inflammatory cascades, generating synergistic pathological intervention [77]. Mitochondrial oxidative stress and inflammation play pivotal roles in the development of sepsis‐associated acute kidney injury (AKI). Cerium oxide nanoparticles (CeNPs), known for their potent and regenerative ROS‐scavenging capacity, have been extensively explored for oxidative stress–related diseases. However, their therapeutic utility is limited by insufficient mitochondrial targeting and aggregation tendencies of ultrasmall nanoparticles. To overcome these challenges, Hui Yu and colleagues developed a ROS‐responsive nanodrug delivery system combining mitochondria‐targeted CeNPs with atorvastatin (Atv/PTP‐TCeria NPs). This system employs triphenylphosphonium‐modified CeNPs (TCeria NPs) coated with a ROS‐sensitive polymer (mPEG‐TK‐PLGA) to achieve controlled drug release. In vitro, Atv/PTP‐TCeria NPs exhibited robust mitochondrial targeting, antioxidant activity, and anti‐apoptotic effects. In a sepsis‐induced AKI mouse model, the nanoparticles preferentially accumulated in the kidneys, preserved mitochondrial integrity, reduced tubular cell apoptosis and necrosis, and effectively alleviated oxidative stress and inflammation. These findings demonstrate the therapeutic potential of ROS‐responsive, mitochondria‐targeted cerium‐based nanoplatforms for sepsis‐associated AKI [78] (Figure 5).

This paradigm—where therapeutic behavior is directly driven by pathological signals—confers a distinct advantage to ROS/RNS‐responsive nanoplatforms, particularly during the inflammatory storm phase, enabling suppression of excessive inflammation while minimizing the risk of systemic immunosuppression [79]. It should be noted, however, that oxidative stress levels vary substantially across sepsis stages and between different organs, providing spatial selectivity while simultaneously imposing stringent requirements on response thresholds and release kinetics [80]. Consequently, recent studies increasingly favor integrating ROS/RNS responsiveness with pH‐ or enzyme‐responsive mechanisms to construct multi‐stimuli–cooperative nanoplatforms with improved precision and safety.

#### 3.1.3 Enzyme‐Responsive Nanoplatforms

During sepsis, the activities of multiple enzymes are markedly altered within the microenvironment of infection foci and injured tissues, a phenomenon consistently documented in both basic and translational studies. Among these, matrix metalloproteinases (MMPs), phospholipase A2 (PLA2), and neutrophil‐associated proteases play critical roles in infection dissemination, barrier disruption, and amplification of inflammatory responses [81, 82]. These pathological features have motivated increasing efforts to exploit aberrant enzymatic activity as endogenous triggers for stimulus‐responsive nanodrug delivery systems [81] (Figure 3C).

The central design principle of enzyme‐responsive nanoplatforms lies in the incorporation of enzyme‐cleavable chemical bonds or peptide sequences into the nanocarrier architecture, enabling structural transformation and drug release in enzyme‐enriched microenvironments [81]. For instance, elevated MMP expression in inflamed and infected tissues has been widely observed, prompting the use of MMP‐sensitive peptide substrates as linkers or protective shells in nanocarriers [83]. Upon exposure to the inflammatory milieu, enzymatic cleavage of these peptides can destabilize the carrier or increase its permeability, thereby facilitating localized drug release. It should be noted, however, that such designs are largely derived from experimental observations in inflammation‐related disease models, and their applicability across different sepsis stages and organ systems remains to be systematically validated [81].

Mahir Mohammed and colleagues developed a biomimetic vancomycin‐loaded solid lipid nanoparticle system (VCM‐AS‐SLNs) to enhance antibacterial efficacy against bacterial sepsis. VCM‐AS‐SLNs exhibited favorable biocompatibility and high affinity for bacterial lipases, which significantly accelerated vancomycin release. Computational simulations and microscale thermophoresis (MST) assays further demonstrated that ascorbyl stearate (AS) and VCM‐AS‐SLNs strongly bind hyaluronidase, inhibiting its enzymatic activity and thereby attenuating pathogenic effects. In vitro antibacterial studies revealed that VCM‐AS‐SLNs reduced the minimum inhibitory concentration against *Staphylococcus aureus*, including methicillin‐resistant strains (MRSA), by twofold compared with free vancomycin, while achieving a fivefold increase in MRSA biofilm eradication. Time–kill assays showed complete bacterial clearance within 12 h, whereas free vancomycin eliminated less than 50% of bacteria after 24 h [84]. Beyond MMPs, PLA2—an enzyme involved in membrane phospholipid metabolism and inflammatory mediator generation—is also dysregulated during sepsis and severe infections, making it another candidate trigger for enzyme‐responsive design [85]. Several studies have employed phospholipid‐ or phospholipid‐mimetic nanocarriers that undergo hydrolysis in the presence of PLA2, thereby inducing carrier disassembly and drug release [86]. While such strategies demonstrate environmental responsiveness in infection‐ and inflammation‐related models, critical issues remain regarding selectivity between host‐ and pathogen‐derived PLA2, as well as carrier stability under complex in vivo conditions. Overall, enzyme‐responsive nanoplatforms do not rely on a single “sepsis‐specific enzyme,” but instead leverage the broadly altered enzymatic landscape characteristic of infection and inflammation to achieve relative selectivity in drug release. Although conceptually sound and experimentally supported, the pronounced spatiotemporal heterogeneity of enzyme expression in sepsis, along with interpatient variability and multi‐organ involvement, suggests that enzyme responsiveness is better suited as a component of multi‐stimulus–integrated systems rather than as a standalone triggering mechanism.

#### 3.1.4 Microbial Stimulus–Responsive Nanoplatforms

Microbial infection constitutes the primary etiological driver of sepsis, and pathogen‐derived molecular signatures play a central role in initiating host immune responses. Pathogens activate the immune system through distinctive molecular patterns, including bacterial endotoxins such as lipopolysaccharide (LPS), lipoteichoic acid (LTA), and microbial DNA or RNA, ultimately triggering systemic inflammatory response [87]. Given the dynamic and systemic nature of sepsis, nanoplatforms responsive to microbial stimuli have emerged as a distinct strategy for precision therapy. Unlike endogenous pathological cues (e.g., pH or ROS), microbial stimulus–responsive nanoplatforms exploit pathogen‐specific molecular signals as triggers, conferring a higher degree of targeting specificity and therapeutic selectivity across different stages of sepsis. However, engineering platforms to engage these microbial cues requires strict navigation of their distinct physicochemical and biological disparities. Specifically, membrane‐bound patternrecognition receptors like lipopolysaccharide (LPS) and lipoteichoic acid (LTA) exhibit high systemic abundance and structural accessibility within the bloodstream during Gram‐negative and Gram‐positive bacteremia, respectively, making them ideal targets for intravascular interception. In contrast, microbial DNA and intracellular enzymes possess low circulatory accessibility, as they are shielded inside the lipid bilayer of viable pathogens, thus requiring nanocarriers to possess active bacterial‐membrane‐penetrating capabilities or wait for pathogen lysis. Furthermore, while secreted virulence toxins and enzymes offer high biological specificity toward localized infection foci, their abundance is highly volatile and strain‐dependent. Within dense bacterial biofilms, severe diffusion barriers dramatically limit the physical accessibility of conventional nanostructures, necessitating the integration of surface charge‐reversal or self‐propelling modalities to effectively penetrate the matrix and engage these cloaked signals.

Among microbial signals, LPS is the most prototypical immunostimulatory molecule associated with Gram‐negative bacterial sepsis [88]. By engaging Toll‐like receptor 4 (TLR4) on host cells, LPS activates downstream NF‐κB and MAPK signaling pathways, inducing robust pro‐inflammatory cytokine production and systemic inflammation [89]. Accordingly, LPS‐responsive nanoplatforms are often designed to sense, capture, or neutralize LPS, thereby enabling targeted intervention at sites of infection or within the circulation. Xianda Liu and colleagues developed a novel blood purification nanoadsorbent platform that addresses two major challenges in sepsis therapy: biofouling‐induced loss of adsorption efficiency and the translational limitations of existing recovery devices. Using electrically neutral phosphocholine‐based ligands to selectively capture LPS, the system leveraged precise charge orientation and dipole rearrangement upon LPS binding to reconcile antifouling performance with high LPS affinity. To enable efficient recovery, the authors engineered a magnetic nanocomposite platform (PCAPAN‐Fe) in conjunction with an extracorporeal LPS‐targeting magnetic array system (ELMAS), ensuring complete nanoparticle retrieval and compatibility with clinical blood purification setups. In a septic rabbit model, this platform achieved 100% survival, 84.7% LPS clearance, and a marked reduction in pro‐inflammatory cytokine levels [90].

**FIGURE 6:**
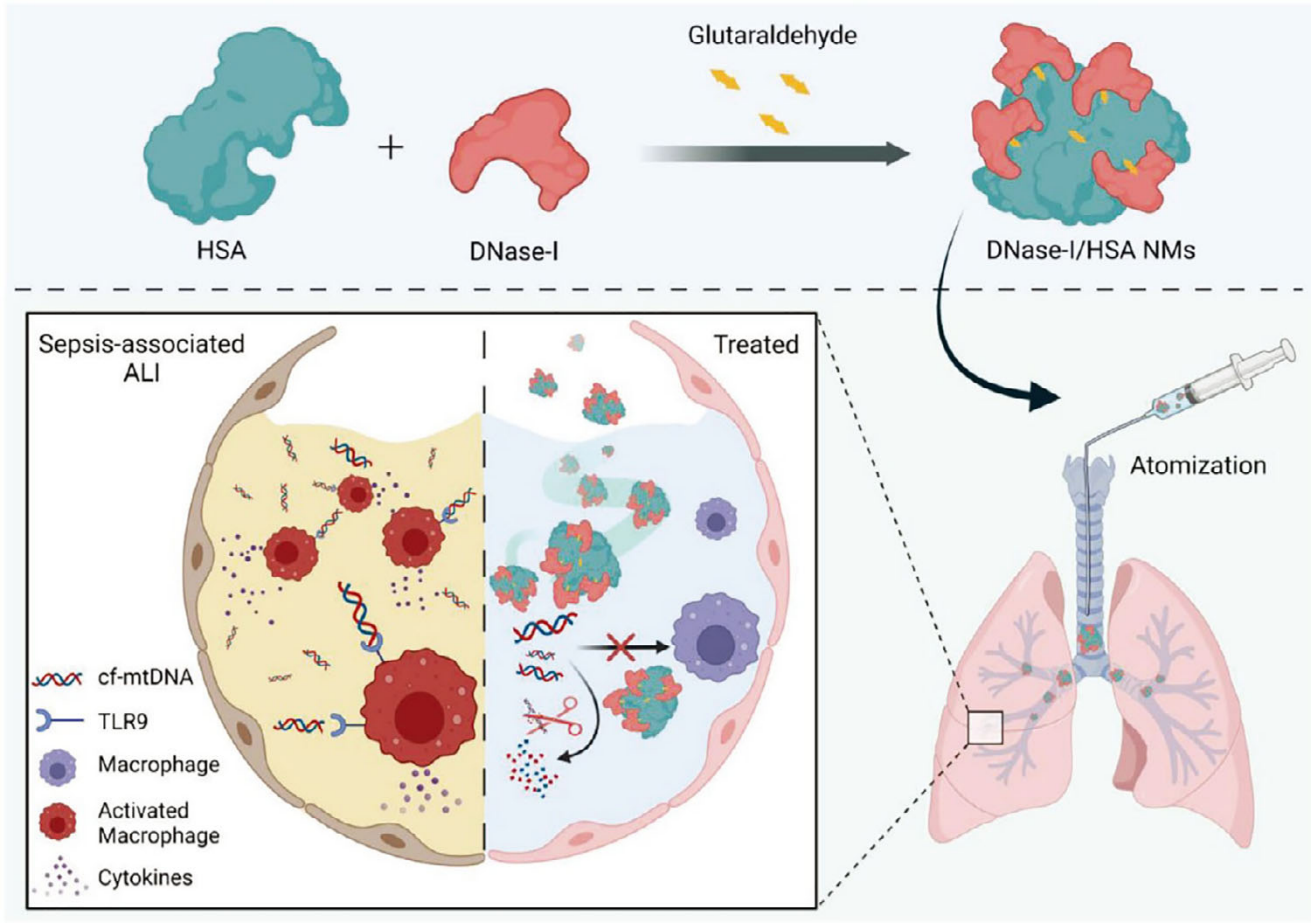
The synthetic procedure of DNase‐I/HSA NMs and their application for sepsis‐associated ALI treating. Fabrication of DNase‐I/HSA NMs via glutaraldehyde‐mediated crosslinking of HSA and DNase‐I (upper panel). After aerosolized intratracheal instillation, self‐propulsive DNase‐I/HSA NMs effectively blocked cf‐mtDNA/TLR9‐mediated alveolar macrophage activation by efficiently scavenging cf‐mtDNA in the pulmonary microenvironment, thereby alleviating sepsis‐associated ALI (lower panel). Reproduced with permission [91]. Copyright 2023, Wiley‐VCH.

Beyond bacterial components, endogenous danger‐associated microbial products can also be exploited as therapeutic targets. Weichang Huang and colleagues identified a pivotal role for cell‐free mitochondrial DNA (cf‐mtDNA) in regulating alveolar macrophage activation during sepsis‐associated acute lung injury (ALI). To address this, they developed a biocompatible hybrid protein nanomotor composed of recombinant deoxyribonuclease I (DNase I) and human serum albumin (HSA), crosslinked via glutaraldehyde to form an inhalable nanotherapeutic platform. The DNase I/HSA nanomotors exhibited self‐propulsion capability, along with superior stability, DNA hydrolytic activity, and biosafety. Pulmonary delivery of these nanomotors effectively cleared cf‐mtDNA from the lungs, attenuated inflammation and lung injury, and significantly improved survival in septic mouse models [91] (Figure 6).

While microbial stimulus–responsive nanoplatforms enable highly precise delivery at infection‐associated sites, potential off‐target exposure in noninfected tissues remains a concern. Further optimization is therefore required to enhance biosafety and minimize unintended effects. Nonetheless, by selectively recognizing pathogen‐derived molecular cues and responding to infection‐specific pathological signals, microbial stimulus–responsive nanoplatforms offer a powerful strategy to improve targeting accuracy and therapeutic precision in sepsis management (Figure 3D).

### 3.2 Exogenous Stimuli

In contrast to endogenous stimuli that exploit pathological microenvironmental cues, exogenous stimulus–responsive nanoplatforms incorporate functional units that can be triggered by external physical signals, enabling spatiotemporal control over drug release [92]. Common exogenous triggers include light, magnetic fields, and ultrasound, offering the theoretical advantage of precise, user‐controlled delivery within a defined therapeutic window [93]. However, for sepsis—a prototypical systemic infectious condition—exogenous stimulus–responsive strategies face practical challenges in terms of clinical accessibility, effective coverage, and safety. Consequently, current investigations are primarily focused on localized infections or specific organ injury models, functioning mainly as complementary or exploratory approaches. Specifically, light‐triggered platforms are fundamentally constrained by poor tissue penetration depth and severe optical scattering in deep organs, while magnetic‐field interventions are hard to implement uniformly across broad systemic circulation without causing localized particulate aggregation or relying on complex, unscalable clinical coil geometries.

Ultrasound, a clinically mature modality with deep tissue penetration, has recently been incorporated into stimulus‐responsive nanoplatforms to achieve controlled, spatiotemporally regulated drug release [94]. Ultrasound‐responsive systems typically rely on cavitation effects or mechanically induced structural changes in the carrier, facilitating drug release and improving local tissue permeability. Compared with light or magnetic stimuli, ultrasound offers the advantages of non‐invasiveness and greater tissue reach, demonstrating translational potential for targeted interventions in organ‐specific injuries [95].

**FIGURE 7:**
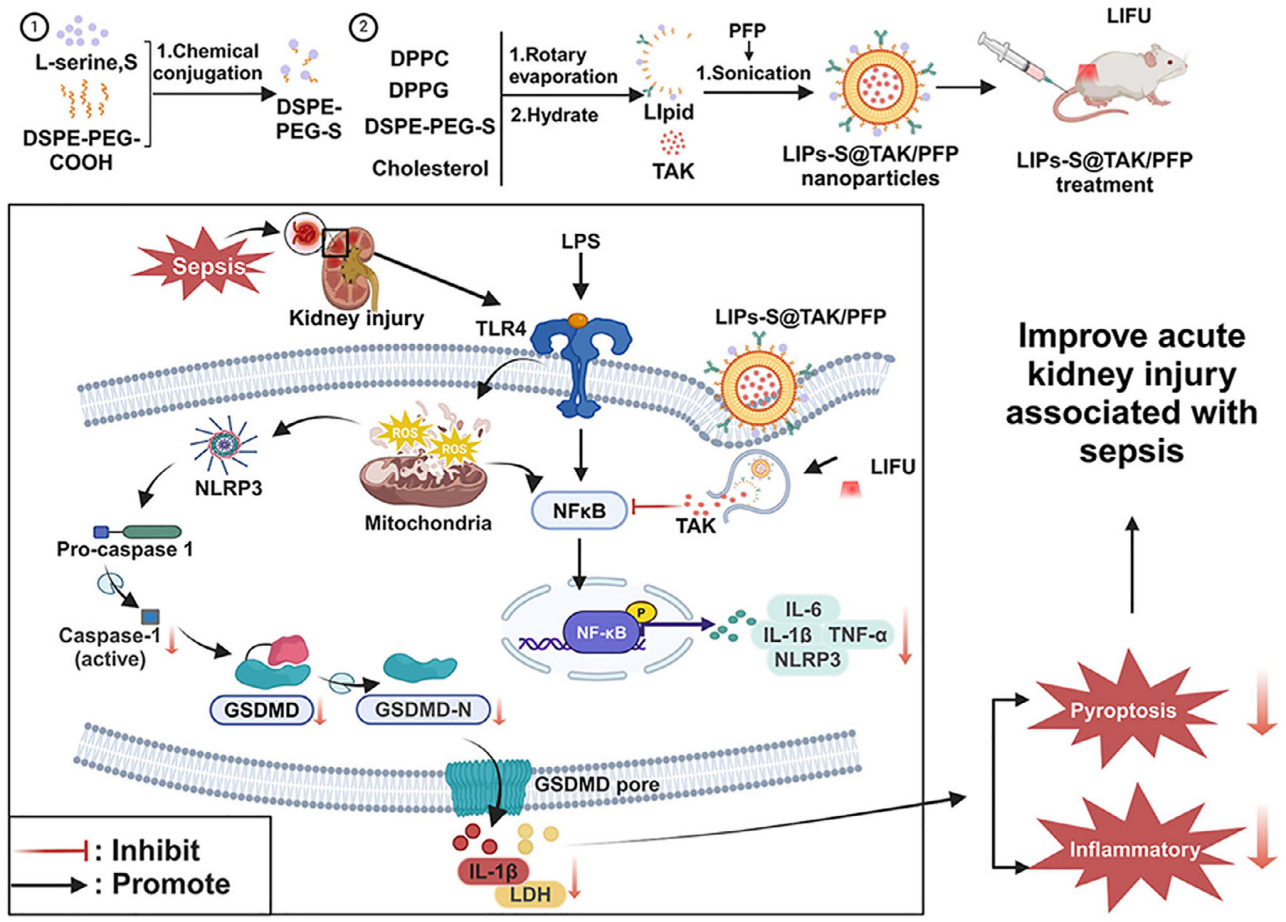
Schematic illustration of the molecular mechanism by which the LIPs‐S@TAK/PFP nanoplatform delivers a TLR4 inhibitor to alleviate SA‐AKI by blocking the NF‐κB signaling pathway and suppressing NLRP3 inflammasome‐mediated pyroptosis. Reproduced with permission [96]. Copyright 2025, Elsevier.

In the context of sepsis‐associated acute kidney injury (SA‐AKI), Yingnan Guo et al. developed an ultrasound‐responsive renal‐targeted nanoplatform by modifying liposomes with serine residues to enhance kidney accumulation. Under low‐intensity focused ultrasound, the system achieved controlled drug release, co‐delivering the Toll‐like receptor 4 (TLR4) inhibitor TAK‐242 along with phase‐change contrast agents for simultaneous therapeutic delivery and imaging. In septic models, this ultrasound‐triggered platform suppressed the TLR4/NF‐κB/NLRP3 inflammatory pathway, reduced pro‐inflammatory cytokine release and oxidative stress, mitigated pyroptosis‐associated injury, and significantly improved renal function and histopathological outcomes. This work represents a compelling example of how exogenous physical stimuli can augment organ‐targeted nanotherapeutic delivery in sepsis [96] (Figure 7).

Jingyi Zhang and colleagues developed an ultrasound‐controlled ROS‐responsive nanoplatform (FOT/Fe_3_O_4_@Lipo‐ICG), in which Fe_3_O_4_ nanoparticles and FOT were encapsulated within ICG‐modified liposomes. Upon ultrasound exposure, Fe_3_O_4_ catalyzed the decomposition of H_2_O_2_ into O_2_ and ˙OH, while FOT reacted with glutathione to generate NO, collectively enhancing antibacterial efficacy. This platform demonstrated potent activity against MRSA‐induced local infections and sepsis, highlighting its potential to augment sonodynamic therapy (SDT) [97].

Overall, while ultrasound‐responsive nanoplatforms offer advantages in organ‐specific targeting and controlled release, their efficacy is contingent on precise application of the external stimulus, limiting coverage across the multi‐organ, dynamically progressing pathology of sepsis. Therefore, at present, ultrasound‐triggered strategies are primarily considered adjunctive tools for enhancing local delivery and organ protection, rather than as core drivers of systemic therapeutic responses. Integrating ultrasound stimulation with endogenous infection‐ or inflammation‐responsive mechanisms to construct multi‐modal, synergistically triggered nanoplatforms may further enhance their translational potential in sepsis therapy. In summary, exogenous stimulus–responsive nanoplatforms enable highly tunable delivery behaviors through external control, providing temporal resolution and release precision that are difficult to achieve with purely endogenous stimuli. Nevertheless, the systemic, dynamic, and multi‐organ nature of sepsis constrains the practical applicability of strategies that rely on localized or directional external stimulation. Consequently, exogenous stimulus–responsive platforms are currently best suited as complementary interventions for localized infection control or specific organ protection rather than as a mainstream approach for systemic sepsis therapy. Recent trends increasingly emphasize the integration of exogenous stimuli with endogenous pathological cues to develop multi‐modal responsive nanoplatforms, aiming to balance spatial accessibility with enhanced control over drug release and safety. This hybrid approach offers a promising conceptual framework for the future optimization of stimulus‐responsive nanotherapeutics in sepsis.

## 4 Stimulus‐Responsive Nanoplatforms for Drug Delivery Strategies

**FIGURE 8:**
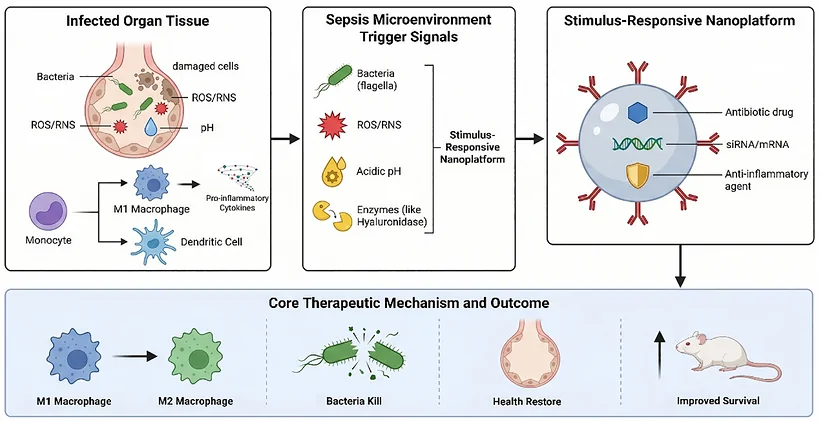
Mechanism of stimulus‐responsive nanoplatforms in the treatment of sepsis‐induced organ injury. The therapeutic approach leverages the specific Sepsis Microenvironment to trigger precision drug release. (Top Left) Infected organ tissue displays pathological hallmarks including bacterial infiltration, accumulation of ROS/RNS, and acidic pH, alongside the differentiation of monocytes into pro‐inflammatory M1 macrophages. (Top Center/Right) The nanoplatform is engineered to respond to specific trigger signals (bacteria, ROS/RNS, low pH, and enzymes like hyaluronidase) to release a synergistic cargo of antibiotics, siRNA/mRNA, and anti‐inflammatory agents. (Bottom) The core therapeutic outcomes include the polarization of M1 macrophages to the anti‐inflammatory M2 phenotype, effective pathogen clearance (Bacteria Kill), and restoration of tissue homeostasis, ultimately leading to improved survival rates in vivo. (Created with BioRender.com).

Stimulus‐responsiveness itself is not a therapeutic endpoint; its ultimate value lies in enabling precise delivery and functional modulation of therapeutic agents. In sepsis—a highly dynamic and systemic disease—selecting appropriate drug types and matching their delivery to disease stage through responsive nanoplatforms is critical for achieving therapeutic efficacy. This section focuses on the application strategies of stimulus‐responsive nanoplatforms for different categories of therapeutics, highlighting their potential to enhance local efficacy, reduce systemic toxicity, and optimize therapeutic windows (Figure 8).

### 4.1 Stimulus‐Responsive Delivery of Antibiotics and Antimicrobial Peptides

Antibiotics remain the cornerstone of sepsis treatment, yet their clinical efficacy is often limited by suboptimal drug concentrations at infection sites, off‐target tissue exposure, and the resulting risks of resistance and systemic toxicity [98]. Stimulus‐responsive nanoplatforms have been proposed as a means to improve delivery rather than replace the antibiotics themselves, optimizing in vivo distribution and release kinetics through environmental triggers [98, 99].

In previous studies, responsive nanocarriers frequently exploit characteristic cues of the infectious or inflammatory microenvironment, such as local acidity, elevated oxidative stress, or enhanced enzymatic activity, to promote antibiotic release at infection foci [99]. This strategy enhances local drug exposure while minimizing premature release in non‐infected tissues, thereby reducing systemic side effects. In sepsis—a lethal infectious disease driven by uncontrolled host immune responses—the infectious microenvironment (IME) is characterized by both increased bacterial load and amplified inflammatory signaling, making it an ideal target for responsive nanotherapeutics. For instance, a bioresponsive nanoplatform targeting the IME was constructed using block copolymers sensitive to both acidic pH and bacterial enzymes. This system self‐assembles into micelles co‐loaded with ciprofloxacin and a small‐molecule anti‐inflammatory agent and is further functionalized with an ICAM‐1 antibody to enhance active targeting to infection sites. In an acute bacterial lung infection model, the nanoplatform achieved “on‐demand” release triggered by local infection‐specific signals, significantly improving drug accumulation and therapeutic efficacy at the site of infection. In a lethal intraperitoneal bacterial challenge model of sepsis, the dual targeting of pathogen clearance and excessive inflammation was shown to markedly improve survival outcomes. This study demonstrates both the feasibility of infection microenvironment‐responsive antibiotic delivery in vivo and the mechanistic potential of “antimicrobial–immune modulation” synergy in nanomedicine‐based sepsis therapy [100]. Beyond conventional antibiotics, antimicrobial peptides (AMPs) have attracted attention due to their diverse mechanisms and potential efficacy against resistant pathogens [101]. However, their in vivo application is hindered by proteolytic degradation and potential cytotoxicity or immune‐related adverse effects at high doses [101, 102]. Stimulus‐responsive nanoplatforms offer a protective and controlled‐release strategy, maintaining stability during circulation and gradually releasing active peptides in infection‐related microenvironments, thereby enhancing both stability and therapeutic window [103]. Overall, the stimulus‐responsive delivery of antibiotics and AMPs does not alter their intrinsic antimicrobial mechanisms but rather regulates the timing and spatial distribution of release to balance efficacy and safety [104]. Current evidence is primarily derived from in vitro and infection‐relevant animal models, and further systematic pharmacodynamic and safety studies are required to validate applicability in the complex systemic environment of sepsis.

### 4.2 Delivery of Anti‐Inflammatory and Antioxidant Agents

Traditional anti‐inflammatory drugs often face spatiotemporal mismatches in systemic administration: premature or excessive intervention may impair host defenses, while insufficient or delayed intervention fails to mitigate excessive inflammation. Stimulus‐responsive nanoplatforms provide a feasible strategy to align anti‐inflammatory or antioxidant drug release with disease progression.

Studies have shown that pH‐, ROS/RNS‐, or enzyme‐responsive nanocarriers can trigger drug release in regions with active inflammation based on characteristic microenvironmental signals. For example, Li Teng et al. developed a pH‐responsive functionalized chitosan nanoplatform co‐loading anti‐inflammatory and antioxidant agents. This system enabled precise release in mildly acidic infectious environments, enhancing both antimicrobial and anti‐inflammatory efficacy while maintaining excellent in vivo biosafety, illustrating the potential of stimulus‐responsive nanoplatforms in precision sepsis therapy [105]. Similarly, Wei He et al. reported an infection microenvironment (IME)‐targeted stimulus‐responsive system for systemic sepsis treatment. In this design, the antimicrobial peptide omiganan and dexamethasone were conjugated via pH‐sensitive hydrazone linkages to form a prodrug, which self‐assembled into nanoparticles coated with hyaluronic acid (HA). HA not only enhanced accumulation in infection sites via ICAM‐1‐mediated endothelial targeting but also facilitated nanoparticle disassembly under hyaluronidase activity, triggering drug release. Acidic conditions cleaved the hydrazone linkages, enabling simultaneous antimicrobial and anti‐inflammatory release. In multi‐tissue infection and sepsis animal models, this strategy effectively eradicated pathogens while suppressing excessive inflammatory responses, significantly improving survival outcomes. This work exemplifies the application of infection microenvironment‐responsive antimicrobial–immune modulation nanotherapy in sepsis [106].

Through this “environment‐driven release” approach, the delivery of anti‐inflammatory and antioxidant drugs can enhance local efficacy and better match therapeutic timing to disease stage. It should be noted, however, that the inflammatory response in sepsis is highly dynamic and spatially heterogeneous; thus, most current data are derived from in vitro or animal studies. Their effectiveness and safety in complex clinical scenarios require further systematic validation. Nevertheless, stimulus‐responsive strategies provide a theoretical framework for coupling drug release more tightly to pathophysiological progression, laying the foundation for staged and individualized interventions in sepsis.

### 4.3 Nucleic Acid and Gene‐Regulating Molecule Delivery

With the rapid advancement of molecular biology and gene‐editing technologies, nucleic acid therapeutics—including siRNA, mRNA, and CRISPR/Cas systems—have garnered increasing attention for sepsis treatment [107, 108]. Compared to conventional small molecules or protein‐based drugs, nucleic acids can precisely modulate inflammatory mediators and key signaling pathways at the transcriptional or genetic level, offering novel possibilities for fine‐tuning immune responses in sepsis. However, their clinical translation is hindered by poor in vivo stability, susceptibility to nuclease degradation, short half‐lives, and low cellular membrane permeability, making effective delivery and functional engagement challenging in the highly dynamic and systemic inflammatory environment of sepsis [109]. These pharmacokinetic and biological barriers significantly limit their therapeutic application.

Yin‐Jin Zhang et al. developed a nanozyme‐based delivery system, DSPE‐TK‐mPEG‐MnO_2_@siSTING (DTmM@siSTING), and characterized it using scanning electron microscopy, dynamic light scattering, nanoparticle size analysis, and gel electrophoresis. Both in vitro ALI cell models and in vivo ALI mouse models demonstrated that DTmM@siSTING possesses robust stability and biocompatibility, effectively delivering siSTING to the lungs. Treatment with DTmM@siSTING significantly reduced ROS levels, myeloperoxidase activity, and pro‐inflammatory cytokine expression, while inhibiting PARP‐1/NLRP3 pathway activation, thereby alleviating sepsis‐induced ALI and associated inflammatory responses [110]. Similarly, Ni Ding et al. constructed a pH‐sensitive nanocarrier system (β‐CD‐PDPA/DSPE‐PEG) to deliver miR‐223 selectively to M1 macrophages. In vitro, the nanoparticles demonstrated excellent pH‐responsiveness, stability, and delivery efficiency. In vivo, NPs/miR‐223 preferentially accumulated at inflamed sites, attenuated inflammation, and improved survival in septic mice by targeting Pknox1 and suppressing NF‐κB pathway activation, thereby modulating macrophage polarization and eliciting anti‐inflammatory effects [73]. Stimulus‐responsive nanoplatforms thus offer a promising strategy for nucleic acid delivery in sepsis, providing environment‐driven, stage‐matched, and cell‐specific release. This lays an important theoretical foundation for precise gene regulation during both hyperinflammatory “cytokine storm” phases and immunosuppressive stages of sepsis.

### 4.4 Delivery of Immunomodulatory Factors and Biological Macromolecules

Immunomodulatory agents—including recombinant cytokines, receptor antagonists, and monoclonal antibodies—as well as other biological macromolecules, have the potential to directly modulate immune signaling pathways and improve pathological outcome [22, 111]. However, their clinical application is often limited by rapid degradation, short half‐life, and systemic administration‐related off‐target effects [111]. Stimulus‐responsive nanoplatforms provide a controllable strategy for delivering immunomodulatory molecules by incorporating environment‐sensitive elements into carrier design [112]. For example, acid‐sensitive, oxidation‐sensitive, or enzyme‐responsive materials can trigger structural changes in the carrier within inflamed or infected regions, facilitating localized release and enhancing both stability and bioavailability [112].

It should be emphasized that most studies to date are confined to in vitro or infectious animal models [113], and their safety and efficacy in clinical sepsis remain to be fully validated. Nonetheless, stimulus‐responsive nanoplatforms offer a conceptual framework for delivering immunomodulators and other biological macromolecules, enabling drug release that balances stability, targeting, and stage‐specific modulation. This approach provides a theoretical basis for future individualized interventions in sepsis.

## 5 Therapeutic Efficacy and Translation Potential of Stimulus‐Responsive Nanoplatforms

Although stimulus‐responsive nanoplatforms present clear conceptual advantages in sepsis treatment, their actual therapeutic efficacy and clinical translation potential must be systematically evaluated [106]. Commonly used animal models of sepsis include cecal ligation and puncture (CLP) and lipopolysaccharide (LPS)‐induced models. These models are designed to mimic key pathological features of sepsis, including the inflammatory storm, immune suppression, and multi‐organ dysfunction [114, 115]. Studies have shown that stimulus‐responsive nanocarriers can target the infectious microenvironment and respond to specific stimuli, effectively intervening in sepsis to reduce systemic inflammation and minimize exposure to non‐target tissues [106].

In terms of safety and biocompatibility, potential concerns include immune toxicity, prolonged retention, and metabolic issues during the translation process [116]. First, to prevent complement activation (e.g., C3a/C5a generation) and rapid clearance by the mononuclear phagocyte system, carriers must maintain excellent blood compatibility, typically achieved via charge‐neutral surfaces or biomimetic cell‐membrane cloaking [117]. Second, to eliminate long‐term retention risks under sepsis‐induced organ dysfunction, platforms must feature predictable biodegradation and metabolic pathways [118]. This is achieved by integrating glutathione‐cleavable bonds (e.g., disulfide linkers) or engineering inorganic matrices to dissociate into renal‐clearable fragments (< 5.5 nm) for efficient excretion [119]. Comprehensive profiling of pharmacokinetics and organ‐specific clearance remains non‐negotiable to prevent chronic tissue toxicity.

Moreover, timing and treatment windows are critical to therapeutic efficacy [120]. Sepsis is highly dynamic, with early‐stage inflammation and late‐stage immune suppression; thus, therapeutic needs vary significantly across stages [121, 122]. Therefore, the design of stimulus‐responsive nanoplatforms should not only focus on local release and targeting but also on adapting to disease progression, enabling dynamic drug delivery and stage‐specific intervention. For example, early intervention should focus on suppressing inflammation and oxidative stress, while later stages should involve delivering immune‐activating factors or gene‐regulating molecules to promote immune function recovery. By optimizing the timing of drug delivery and response design, nanoplatforms can maximize therapeutic window compatibility, providing both theoretical and experimental support for clinical translation in sepsis. In summary, these responsive mechanisms are not scattered events but form a unified therapeutic cascade matching sepsis progression. In the early phase, nanoplatforms execute upstream cGAS‐STING inhibition to achieve rapid cytokine suppression, while simultaneously shifting M1 macrophage polarization into a tissue‐repairing M2 phenotype. As sepsis advances, the system pivots to alleviate T‐cell exhaustion, thereby reversing immune paralysis. By integrating myeloid reprogramming with lymphocyte reactivation, these smart carriers systematically drive host immune recovery.

## 6 Current Challenges and Future Development Directions

Despite the conceptual advantages of stimulus‐responsive nanoplatforms in sepsis treatment, their clinical translation faces several challenges. First, the pathological stimuli in sepsis exhibit high individual variability. Different patients have significant differences in inflammation intensity, immune suppression, and metabolic state, which impose higher demands on the precise release of stimulus‐responsive carriers [123, 124]. Existing carriers are often designed based on average pathological features, making it difficult to fully capture dynamic changes across patients, potentially resulting in inconsistent efficacy or limited benefit for some individuals.

Despite remarkable progress in stimulus‐responsive nanoplatforms for sepsis management, several critical challenges remain before clinical translation can be realized. First, there is an inherent translational gap between animal models and clinical sepsis. Although CLP and LPS‐induced models can effectively reproduce inflammatory storms, immune suppression, and certain aspects of organ injury, they fail to fully recapitulate the heterogeneity of human sepsis, including dynamic immune trajectories, complex multi‐organ interactions, and individualized disease progression, which limits the direct translation of experimental findings into clinical practice [125]. Future studies should therefore focus on optimizing animal models, integrating patient‐derived systems, and developing more clinically relevant multi‐organ in vitro platforms to improve the predictive accuracy of nanotherapeutic evaluation. Second, the current development stage of stimulus‐responsive nanoplatforms also reveals a significant gap between relatively mature single/dual stimulus‐responsive systems and more sophisticated intelligent therapeutic platforms. To date, single‐ or dual‐stimulus‐responsive systems, such as pH‐sensitive hydrazone bond cleavage, ROS‐responsive antioxidant components, and enzyme‐responsive peptide shells, have been extensively validated in preclinical CLP and LPS models, demonstrating enhanced local drug accumulation and reduced systemic toxicity. In contrast, complex closed‐loop therapeutic systems integrating real‐time biosensing, AI‐driven decision‐making, and phenotype‐specific adaptive drug release remain largely at the conceptual stage. Their clinical translation requires overcoming major engineering challenges, including signal recognition accuracy, signal transduction fidelity, dynamic response regulation, and long‐term biological stability under conditions of severe inflammation and multi‐organ dysfunction.

Additionally, large‐scale and standardized production remains a critical bottleneck for the clinical application of multifunctional nanoplatforms [112]. The complexity of functional design, multi‐stimulus responsiveness, and bioactive drug loading increases manufacturing difficulty and may compromise batch‐to‐batch consistency. Therefore, the establishment of controllable, high‐throughput, and quality‐assured manufacturing processes is essential to ensure reproducibility, biocompatibility, and clinical feasibility. Moreover, systematic evaluation of the in vivo fate, including biodistribution, degradation behavior, clearance pathways, and long‐term biosafety, remains indispensable before clinical translation [18].

**FIGURE 9:**
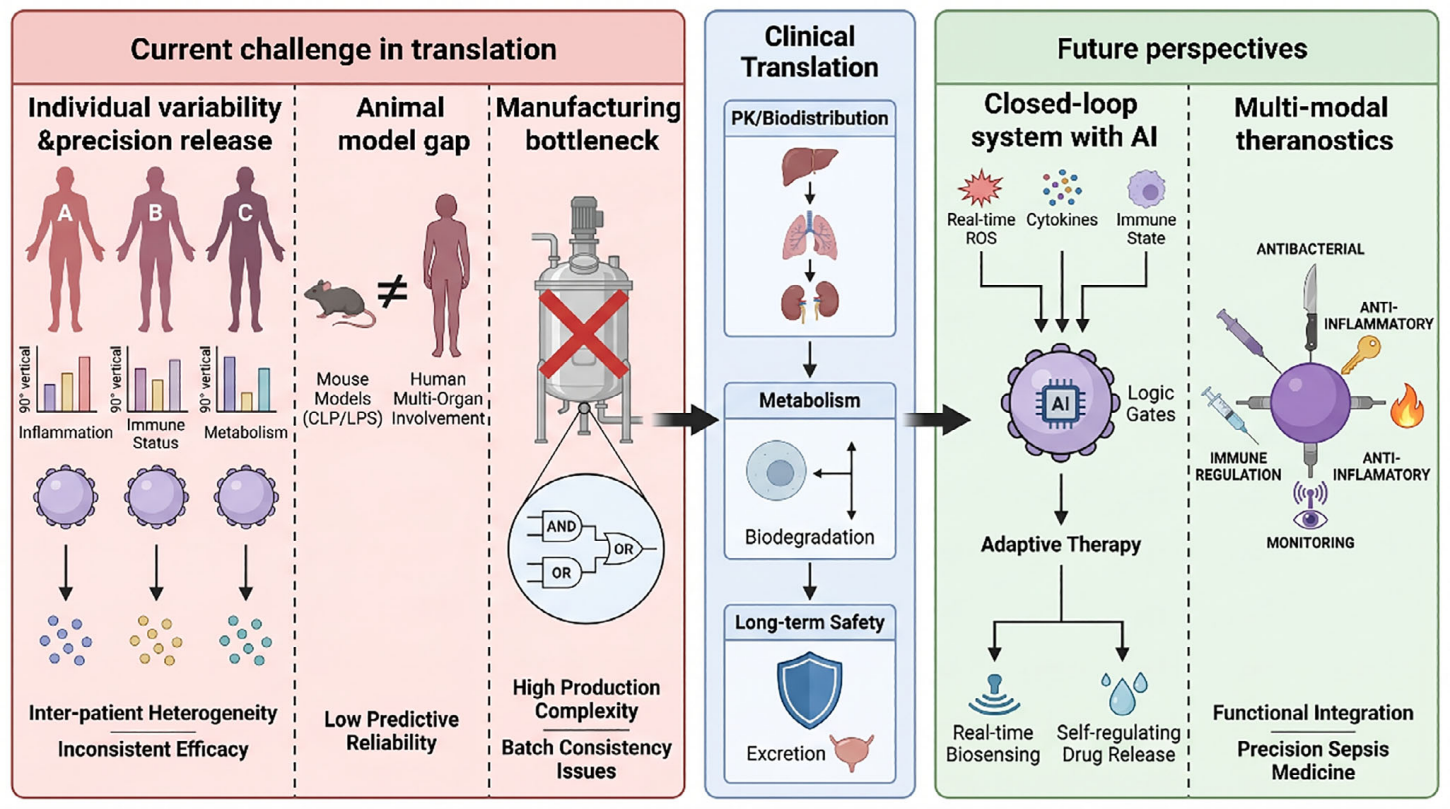
Overcoming clinical translation barriers in sepsis‐responsive nanomedicine. This schematic summarizes the transition from current obstacles to future innovations in sepsis therapy. (Left) Current Challenges include individual variability (patient heterogeneity and inconsistent efficacy), the animal model gap (low predictive reliability of CLP/LPS models), and manufacturing bottlenecks (high production complexity and batch consistency issues). (Center) The Clinical Translation Bridge emphasizes the critical roles of PK/biodistribution, metabolic profiling, and long‐term safety assessment in advancing nanoplatforms. (Right) Future Perspectives highlight the development of closed‐loop AI systems for real‐time biosensing and adaptive therapy, alongside multi‐modal theranostics—visualized as a “Swiss army knife” nanoplatform—to achieve functional integration and precision sepsis medicine. (Created with BioRender.com).

Looking ahead, integrating real‐time biosensing and artificial intelligence into closed‐loop response systems may provide new opportunities for personalized sepsis therapy. Beyond functioning as regulatory components within therapeutic feedback networks, AI‐driven algorithms may further accelerate the upstream design of nanomaterials by establishing quantitative relationships between material structures, molecular descriptors, and biological responses. Through machine learning‐assisted high‐throughput screening, it may become possible to predict stimulus‐responsive behaviors, optimize nanostructural configurations, and identify candidate formulations tailored to specific sepsis endotypes. Furthermore, the development of multimodal theranostic nanoplatforms integrating antibacterial activity, immunomodulation, antioxidant protection, and real‐time monitoring capabilities may enable dynamic adaptation to disease evolution. Such intelligent systems have the potential to optimize therapeutic timing, minimize systemic toxicity, and facilitate the transition from conventional empirical treatment toward precision‐guided sepsis management (Figure 9). In conclusion, although stimulus‐responsive nanoplatforms show great potential in conceptual and experimental research, their clinical translation faces challenges related to individual differences, model applicability, manufacturing processes, and dynamic response control. By integrating nanomaterials, immunology, sensing technologies, and artificial intelligence, future developments may enable stage‐specific, personalized, and multi‐modal treatments for sepsis, significantly improving patient outcomes and advancing the field of precision medicine.

## 7 Conclusion and Future Outlook

Stimulus‐responsive nanoplatforms, by sensing sepsis‐specific pathological signals, enable precise drug release and immune modulation, demonstrating unique advantages in suppressing the inflammatory storm, reshaping innate and adaptive immune functions, and restoring immune homeostasis. Compared to conventional treatments, these platforms offer a combination of stage‐specific intervention, localized accumulation, and systemic safety, providing new strategies for precise drug delivery and immune homeostasis reconstruction in sepsis. Looking forward, the integration of real‐time biosensing, multi‐modal therapy, and artificial intelligence‐assisted adaptive platforms is poised to push stimulus‐responsive nanocarriers toward clinical translation. This will enable individualized, stage‐specific sepsis treatments, ultimately improving patient prognosis and driving the development of precision medicine.

## Author Contributions

**Y.L**.: conceptualization, investigation (literature search), visualization, Writing – original draft. **K.W**.: conceptualization, investigation (literature search), data curation, writing – original draft. **Z.X**.: investigation, visualization, writing – review & editing. **X.Z**.: investigation, data curation, writing – review & editing. **Z.L**.: conceptualization, visualization, writing – review & editing. **X.B**.: investigation, resources, writing – review & editing. **Y.W**.: conceptualization, supervision, project administration, writing – review & editing, funding acquisition.

## Funding

This study was supported by grants from the National Natural Science Foundation of China (Nos. 82002101, 82002096), Hubei Provincial Natural Science Foundation of China (No. 2023AFB825), and Tongji Hospital, Tongji Medical College, Huazhong University of Science and Technology (No. 2023A15).

## Conflicts of Interest

The authors declare no conflicts of interest.

## Data Availability

The data that support the findings of this study are available from the corresponding author upon reasonable request.
